# Sex as a Risk Factor for Solar Ultraviolet Radiation Exposure?–Dosimetry in Danish Outdoor Workers

**DOI:** 10.1111/php.13317

**Published:** 2020-08-28

**Authors:** Helene Borup, Ole Steen Mortensen, Kasper Grandahl

**Affiliations:** ^1^ Department of Occupational and Social Medicine Holbaek Hospital Part of Copenhagen University Hospital Holbaek Denmark; ^2^ Section of Social Medicine Department of Public Health University of Copenhagen Copenhagen Denmark

## Abstract

Solar ultraviolet radiation (UVR) exposure is a known risk factor for the development of skin cancer. Heterogeneity in solar UVR exposure may explain the diversity in skin cancer incidence between men and women. This, however, has not previously been investigated in Danish outdoor workers using UVR dosimetry. The aim of this study was to evaluate sex differences in solar UVR dosimetry in Danish outdoor workers on working and leisure days. A cross‐sectional design was used to collect dosimetry data during the Danish summer season (May to September). Analysis was based on an electronic questionnaire and dosimetry data from 450 outdoor workers (88 women, 362 men). Dosimetry data were reported as standard erythema dose (SED). The daily median SED (Interquartile range) on working days was 1.6 (2.5) in men and 1.5 (2.1) in women while on leisure days it was 0.5 (1.4) in men and 0.6 (1.3) in women. Analysis by multiple linear regression did not show any association between daily median SED and sex on either working or leisure days. In conclusion, solar UVR exposure in Danish outdoor workers did not vary according to sex.

## Graphical abstract

Objective measurements from 450 Danish outdoor workers wearing personal dosimeters showed no sex difference in solar ultraviolet radiation (UVR) exposure on either working days or leisure days. However, the data found high levels of solar UVR exposure on working days in both men and women with the consequent risk of developing work‐related skin cancer.

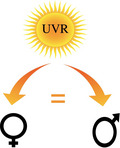

## Introduction

Solar ultraviolet radiation (UVR) is classified as a Group 1 human carcinogen by the International Agency for Research on Cancer (1). The World Health Organization has assessed the fraction of global disease burden attributable to solar UVR exposure to be between 50–90% for both cutaneous malignant melanoma (CMM) and basal cell carcinoma (BCC) and between 50–70% for squamous cell carcinoma (SCC) (2).

In Denmark, campaigns to prevent excess solar UVR exposure have predominantly targeted the general population and to a lesser extent outdoor workers specifically (3). Nevertheless, a recent dosimetry study found worrying levels of solar UVR exposure in Danish outdoor workers on working days (4).

The majority of outdoor workers are men, however, in the Danish building and construction industry, and agriculture and fishing industries, where outdoor work is common, an estimated 11‐33% workers are women (5). As such, the risk of developing work‐related skin cancer applies to both sexes.

The occurrence of skin cancer in Denmark has increased markedly compared to the 1970s. Between 1978 and 2007, the age‐standardized incidence rate (per 100 000 person‐years) for BCC increased from 27.1 to 96.6 in women and from 34.2 to 91.2 in men. The SCC incidence increased in the same period from 4.6 to 12.0 cases per 100 000 person‐years for women and 9.7 to 19.1 cases for men (6).

In Denmark, CMM is more common in women than in men (28.9 cases *vs* 22.7 cases per 100,000 person‐years) excluding the 60+ age group where the incidence is higher among men (7). In 2018, Danish women were estimated to have the highest global incidence rate of CMM with 33.1 cases per 100 000 population (age‐standardized) (8).

Sex differences in solar UVR exposure may explain the differences in skin cancer incidence between men and women. This, however, has only been studied to a limited extent by use of dosimetry, and mainly in studies limited to particular subgroups of the population. These studies found either no sex difference or higher levels of solar UVR exposure in men compared to women in the general population (9, 10, 11, 12, 13, 14, 15, 16). Conversely, the only previous dosimetry study to have investigated sex difference in solar UVR exposure in outdoor workers (farmers) found higher levels of solar UVR exposure in women compared to men (17).

There is somewhat of a gap in the literature when it comes to dosimetry studies on sex difference in solar UVR exposure. This is especially true in outdoor workers, where sex differences in solar UVR exposure might reflect differences in both behavior and skin cancer incidence between men and women that could influence future strategies for primary skin cancer prevention in the working environment. The overall aim of this study was to study sex difference in solar UVR dosimetry in a Danish working population.

## Materials and Methods

This study is a secondary analysis of dosimetry data originally reported by Grandahl et al. (4), as part of the PhD project: *Solar Ultraviolet Radiation Exposure, Sun Protection Behavior and Skin Photodamage in Danish Workers* (18). For a more detailed description on the original data collection and process, we kindly refer to the previously published article (4) while a short summary is given below.

### Study design

The original study was a cross‐sectional study of solar UVR exposure on working and leisure days in a Danish working population.

### Recruitment

Nationwide recruitment was carried out between April 2016 and May 2017. Danish unions, municipalities and company health and safety organizations supported and facilitated the recruitment process through information via e‐mail, journals, notice boards and meetings. It was estimated that several thousand workers were invited to participate. Hereof, five hundred and thirty‐one workers from more than 50 different worksites responded to the invitation. All respondents went through a screening process conducted by e‐mail questionnaire and telephone interview.

Inclusion criteria were various job titles, including mainly outdoor work, equal‐parts‐outdoor‐and‐indoor work and mainly indoor work, and participants should either hold a permanent or trainee position.

Exclusion criteria were insufficient Danish language skills, retirement or sick leave.

### Study population

Five hundred and fifteen participants were enrolled in the study after fulfilling the inclusion and exclusion criteria. In this secondary analysis, we excluded mainly indoor workers and our study population consisted of 450 mainly outdoor or equal‐parts‐outdoor‐and‐indoor workers. Hereof a subgroup of 78 gardeners (41 women).

### Data collection and management

The data were collected, using objective measurements of solar UVR exposure and an electronic study questionnaire. The study had one hundred and two personal UV‐B dosimeters available for measurements, each set to measure solar UVB at 10‐s intervals between 7 AM and 7 PM. Data collection was carried out between May and September 2016 and in April and May 2017 with the intent to distribute the collection of dosimetry data somewhat equally across the summer season with respect to participant occupation, while taking into account participant vacation plans and the limited number of dosimeters available. Each individual would carry out measurements on working days, leisure days or both. The median number of days measured was 10 working days and 4 leisure days. The International Commission on Illumination (CIE) action spectrum weighted dosimeter data were converted to daily standard erythema doses (SED). Data collected by daily SMS text messages were used to differentiate between working and leisure days and ensure that the dosimeter was worn uncovered on the wrist or forearm throughout the daily measurement period. Items in the electronic questionnaire provided demographic data as well as self‐reported data on job title, education, lifestyle, sun behavior and family history of skin cancer. Most variables were categorical with two or more levels. In this secondary analysis, multilevel variables were binary coded to increase the number of observations in each group. The categorical variables used in this secondary analysis were as follows:

Outdoor work (mainly outdoor work *vs* equal‐parts‐outdoor‐and‐indoor work), smoking (never smoker *vs* current/former smoker), alcohol (no alcohol consumption *vs* one or more unit alcohol per week), higher education (no higher education of minimum 2 years duration *vs* higher education of minimum 2 years duration), history of sunburn at work (never *vs* often/rarely), use of sun protection at work (never/rarely use of any *vs* always/often use of any), use of sun protection at leisure (never/rarely use of any *vs* always/often use of any), family history of skin cancer (none/don't know if skin cancer in family *vs* skin cancer in family), exercise (no exercise once or more per week *vs* exercise once or more per week).

### Measurement

In this secondary analysis, daily median SED on working and leisure days was determined for each participant.

### Statistical analysis

Descriptive analysis using histograms, Kolmogorov–Smirnov test and Q–Q plots were used to check for normal distribution. As data were not normally distributed the median and interquartile range (IQR) were used to describe the daily median SED. Chi‐square test was used to examine associations between categorical variables, while one‐way ANOVA was used to examine the difference between groups for the continuous variable (age). Natural log (ln) transformation of the dependent variable was used to obtain a reasonable normal distribution of residuals and linear regression analysis was carried out. The daily median SED was the dependent variable, while outdoor work, sex, age, smoking, alcohol, exercise, education, sunburn at work, sun protection at work, sun protection at leisure and family history were independent variables. In the final multivariate analysis, only independent variables with *P* < 0.1 were included. We conducted a forward multiple linear regression analysis on both working and leisure days, and in a subgroup of gardeners. Statistical significance was determined using *α* = 0.05. IBM SPSS version 21 was used for data analysis.

## Results

### Overview of collected data

410 workers (81 women) on working days and 403 workers (81 women) completed dosimetry on leisure days. The median number of days (IQR) with measurements was 10 (3) for men and 10 (2) for women. On leisure days the median number was 4 (3) for men and 4 (2) for women. The most common occupations were gardener (*n* = 83, 42 women), postal worker (*n* = 44, 18 women), unskilled laborer (*n* = 47, 8 women), carpenter (*n* = 40, 1 women), roofer (*n* = 39, 0 women) and road worker (*n* = 31, 1 women).

Table 1 compares the distribution of independent variables by sex and shows a statistically significant sex difference for alcohol consumption (*P* < 0.05).

**Table 1:** Summary descriptive analysis of independent variables, including the distribution by sex and sex difference

	Male, *N* (% of total)	Female, *N* (% of total)	*P*‐value
Participants			
Sex	362 (80%)	88 (20%)	
Age			
Median (IQR)	47 (18) years	47 (11) years	0.675
Higher education (≥2 years)			
No	292 (82%)	68 (77%)	0.260
Yes	62 (18%)	20 (23%)	
Outdoor work			
Mainly outdoor	311 (86%)	77 (88%)	0.698
Equal‐parts‐indoor‐outdoor	51 (14%)	11 (12%)	
Smoking status			
Never	191 (54%)	50 (57%)	0.629
Former/current	163 (46%)	38 (43%)	
Alcohol (≥1 unit per week)			
No	58 (16%)	24 (27%)	0.019*
Yes	296 (84%)	64 (73%)	
Exercise (≥1 per week)			
No	165 (47%)	42 (48%)	0.851
Yes	189 (53%)	46 (52%)	
Sunburn at work			
No	38 (11%)	9 (10%)	0.890
Yes	316 (89%)	79 (90%)	
Sun protection at work			
Rarely/never	112 (32%)	34 (37%)	0.212
Always/often	242 (67%)	54 (63%)	
Sun protection at leisure			
Rarely/never	114 (32%)	23 (26%)	0.271
Always/often	240 (68%)	65 (74%)	
Family history of skin cancer			
No/don't know	303 (85%)	80 (92%)	0.095
Yes	53 (15%)	7 (8%)	

IQR, Interquartile Range.

* Statistically significant *P*‐value.

### Solar UVR exposure on working days in men and women

The daily median SED (IQR) was 1.6 (2.5) in men and 1.5 (2.1) in women.

Multiple linear regression analysis did not show any association between daily median SED and sex. The analysis showed a statistically significant negative association between daily median SED and working equal‐parts‐outdoor‐and‐indoor while a statistically significant positive association was found between daily median SED and drinking alcohol and having a family history of skin cancer. The final model could explain 8.1% of the variation in the daily median SED. The results are shown in Table 2.

**Table 2:** Results of the multiple linear regression models for daily median SED on working days

		β (*P*‐value)
Model 1		
*R* ^2^ (*P*‐value)	**0.057 (<0.001)** ^*^	
Age^†^		−0.007 (0.884)
Sex^‡^		−0.035 (0.478)
Working equal‐parts‐outdoor‐and‐indoor^§^		−**0.236 (<0.001)** ^*^
Model 2		
*R* ^2^ (*P*‐value)	**0.071 (<0.001)** ^*^	
Age^†^		−0.006 (0.907)
Sex^‡^		−0.022 (0.644)
Working equal‐parts‐outdoor‐and‐indoor^§^		−**0.244 (<0.001)** ^*^
Alcohol ≥ 1 unit per week^||^		**0.121 (0.014)** ^*^
Model 3		
*R* ^2^ (*P*‐value)	**0.081 (<0.001)** ^*^	
Age^†^		0.008 (0.867)
Sex^‡^		−0.018 (0.719)
Working equal‐parts‐outdoor‐and‐indoor^§^		−**0.247 (<0.001)** ^*^
Alcohol ≥ 1 unit per week^||^		**0.104 (0.034)** ^*^
Family history of skin cancer^¶^		**0.102 (0.036)** *

SED, standard erythemal dose.

* Statistically significant *P*‐value.

^†^ Yearly change.

^‡^ Referent group = male.

^§^ Referent group = mainly outdoor work.

^||^ Referent group = no alcohol consumption per week.

^¶^ Referent group = no/don't know family history of skin cancer.

Additional analysis using each month as a variable did not affect the results (data not shown).

### Solar UVR exposure on leisure days in men and women

The median SED (IQR) was 0.5 (1.4) in men and 0.6 (1.3) in women.

Multiple linear regression did not show any association between daily median SED and sex. The analysis showed a statistically significant positive association between daily median SED and higher age, having more than 2 years of higher education, drinking alcohol and exercising at least once a week.

The final model could explain 7.3% of the variation in the daily median SED. The results are shown in Table 3.

**Table 3:** Results of the multiple linear regression models for daily median SED on leisure days

		β (*P*‐value)
Model 1		
*R* ^2^ (*P*‐value)	**0.036 (0.001)** *	
Age^†^		**0.189 (<0.001)** *
Sex^‡^		0.017 (0.727)
Model 2		
*R* ^2^ (*P*‐value)	**0.049 (<0.001)** *	
Age^†^		**0.186 (<0.001)** *
Sex^‡^		0.012 (0.813)
Higher education ≥ 2 years^§^		**0.115 (0.02)** *
Model 3		
*R* ^2^ (*P*‐value)	**0.060 (<0.001)** *	
Age^†^		**0.186 (<0.001)** *
Sex^‡^		0.021 (0.434)
Higher education ≥ 2 years^§^		**0.113 (0.022)** *
Alcohol ≥ 1 unit per week^||^		**0.102 (0.039)** *
Model 4		
R^2^ (*P*‐value)	**0.073 (<0.001)** *	
Age^†^		**0.187 (<0.001)** *
Sex^‡^		0.025 (0.608)
Higher education ≥ 2 years^§^		**0.104 (0.035)** *
Alcohol ≥ 1 unit per week^||^		**0.108 (0.028)** *
Exercise ≥ once a week^¶^		**0.113 (0.021)** *

SED, standard erythemal dose.

* Statistically significant *P*‐value.

^†^ Yearly change.

^‡^ Referent group = male.

^§^ Referent group = no higher education of minimum 2 years duration.

^||^ Referent group = no alcohol consumption per week.

^¶^ Referent group = no exercise once or more per week.

Additional analysis using each month as a variable did not have a significant effect on the results (data not shown).

### Solar UVR exposure on working days and leisure days in gardeners

On working days, the subgroup analysis did not show any association between daily median SED and sex but showed a statistically significant positive association between daily median SED and alcohol consumption (*β* = 0.343, *P* = 0.002, data not shown).

On leisure days, the subgroup analysis did not show any association between daily median SED and sex, but showed a statistically significant positive association between daily median SED and higher age (*β* = 0.367, *P* = 0.001, data not shown).

## Discussion

Overall, no association was found between sex and solar UVR exposure in Danish outdoor workers on either working days or leisure days. This was also true among gardeners, with an almost equal distribution of men and women. In fact, only 0.1 SED difference in solar UVR exposure was found between men and women on both working and leisure days.

Comparing our results with the relatively few previous solar UVR dosimetry studies is somewhat difficult as the population, climate, dosimeter anatomical position and measurement properties vary widely between studies. In one Australian study, personal solar UVR dosimetry during a weekday and a weekend day in summer and winter showed a higher proportion of ambient solar UVR exposure in men compared to women (3.9% *vs* 1.6% in summer and 9.0% *vs* 4.9% in winter) (12). In US radiologic technologists, individual dosimetry on both working and leisure days showed a minor but statistically significant sex difference in daily median solar UVR exposure (0.95 SED in men and 0.71 SED in women, *P* = 0.01) (11). In Austrian farmers, mean daily solar UVR exposure on working days was higher among women compared to men (3.65 SED *vs* 2.07 SED, *P* < 0.05) (17). A Danish observational dosimetry study, of a random sample of Danes, found a small sex difference in daily median solar UVR exposure at leisure (1.4 SED in men and 1.1 SED in women) (13). In five other Danish studies, no sex difference was found in solar UVR exposure on working days or on leisure days (9, 10, 14, 15, 16). As such, the results in our study are in good agreement with the results in most previous Danish studies, namely that there is little or no sex difference in solar UVR exposure. Also, the finding of no sex difference on working days in our study is not surprising, since men and women with outdoor work are likely to have largely the same working hours and job tasks.

Even so, risk behavior in the form of sunbathing and/or outdoor exposure of shoulders or upper body is a major risk factor for solar UVR exposure and has been shown to be more common in women compared to men (9, 10, 14, 15, 16). The Danish Cancer Society has shown that sun protection behavior in the general Danish population also varies by sex; women sunbathe more than men do and are better at seeking shade and using sunscreen than men, while men are better covering up using sunhats and clothes than women (3). A recent German study among outdoor workers found that women used sunscreen and sunglasses more frequently and had better opportunity to stay in the shade compared with men, while men had a tendency to cover their heads more often than women (19). It is not known whether there is a sex difference in the, generally inadequate, use of sun protection by Danish outdoor workers (20).

In this study, no association was found between the use of sun protection and solar UVR exposure on either working or leisure days.

Our study found, for working days, a statistically significant association between mainly outdoor work and daily median SED while it showed a statistically significant positive association with alcohol consumption and having a family history of skin cancer. On leisure days, a significant positive association was found between higher age, higher education with a duration ≥ 2 years, exercising once a week and daily median SED.

It is not surprising that mainly outdoor workers are exposed at higher levels of daily median SED compared to equal‐parts‐outdoor‐and‐indoor workers. Drinking alcohol might lead to a more general risk behavior and people exercising may be more exposed during outdoor sports. The association between age and solar UVR exposure shows mixed results from other Danish studies as one study (13), like ours, found a positive association, while two other studies only found a statistically significant association with increasing age in people younger than 20 years (9, 14). Only one other Danish study reported specifically on the association between educational level and solar UVR exposure, finding no association between the two in regression analysis (13). Our finding that having a family history of skin cancer was positively associated with daily median SED may reflect some kind of inherited work‐related behavior within families.

Finally yet importantly, this study shows that Danish outdoor workers exceed the threshold limit for solar UVR exposure recommended by the International Commission on Non‐Ionizing Radiation Protection of 1.0–1.3 SED for an 8‐h work period (21) and therefore may be at risk of developing work‐related skin cancer regardless of sex.

### Strengths and limitations

A major strength of this study is the use of dosimetry data from a group of 450 participants from which data was collected from 410 participants on working days and 403 on leisure days, and a median number of measurement days per participant of 10 and 4, respectively. The representativeness of the results was further improved by the decision to collect dosimetry data for shorter periods in a relatively large study population, as opposed to longer periods in a smaller study population.

The representation of 20% women in the study population corresponds reasonably well with the estimated sex distribution of 11–33% women in Danish outdoor workplaces (5) and is also reasonably reflected by data collection in each month where the representation of women varies from 13% to 26%.

The risk of selection bias due to convenience sampling cannot be ruled out. The risk of information bias due to sun behavior change associated with wearing the dosimeter is also possible. In both cases, however, the risk must be assumed the same for men and women.

Data collection using daily SMS text messages served to distinguish dosimetry on working and leisure days, but not working and leisure hours. This may constitute a study limitation on working days, more so than on leisure days. However, as stated in the discussion, it is unlikely that the working hours and leisure hours differ significantly between men and women on working days.

## Conclusion

Solar UVR exposure in Danish outdoor workers did not vary according to sex, neither on working days nor on leisure days. However, Danish outdoor workers are exposed to high levels of solar UVR on working days with the consequent risk of developing work‐related skin cancer and should be the target of preventive efforts regardless of sex.
